# Voices from the Patients: A Qualitative Study of the Integration of Tuberculosis, Human Immunodeficiency Virus and Primary Healthcare Services in O.R. Tambo District, Eastern Cape, South Africa

**DOI:** 10.3390/idr15020017

**Published:** 2023-03-06

**Authors:** Ntandazo Dlatu, Kelechi Elizabeth Oladimeji, Teke Apalata

**Affiliations:** 1 Department of Public Health, Faculty of Health Sciences, Walter Sisulu University, Private Bag X1, Mthatha 5117, South Africa; 2 College of Graduate Studies, University of South Africa, Johannesburg 0001, South Africa; 3 Department of Laboratory Medicine and Pathology, Faculty of Health Sciences and National Health Laboratory Services (NHLS), Walter Sisulu University, Private Bag X1, Mthatha 5117, South Africa

**Keywords:** TB-HIV integration, challenges and barriers, patients, O.R. Tambo district, Eastern Cape, South Africa

## Abstract

Tuberculosis (TB), a disease of poverty and inequality, is a leading cause of severe illness and death among people with human immunodeficiency virus (HIV). In South Africa, both TB and HIV epidemics have been closely related and persistent, posing a significant burden for healthcare provision. Studies have observed that TB-HIV integration reduces mortality. The operational implementation of integrated services is still challenging. This study aimed to describe patients’ perceptions on barriers to scaling up of TB-HIV integration services at selected health facilities (study sites) in Oliver Reginald (O.R) Tambo Municipality, Eastern Cape province, South Africa. We purposely recruited twenty-nine (29) patients accessing TB and HIV services at the study sites. Data were analyzed using qualitative content analysis and presented as emerging themes. Barriers identified included a lack of health education about TB and HIV; an inadequate counselling for HIV and the antiretroviral drugs (ARVs); and poor quality of services provided by the healthcare facilities. These findings suggest that the O.R. Tambo district needs to strengthen its TB-HIV integration immediately.

## 1. Introduction

Because of disease-specific funding or service delivery strategies, early on in the HIV epidemic, HIV services were offered through vertical programs [1,2]. However, segregated service delivery was believed to lead to patients needing to visit multiple facilities for their various health requirements or problems, or having to visit the same facility on different days of the week [3,4,5]. As a result, there were “lost chances” to meet the demands of patients in terms of holistic treatment [1,2]. It also resulted in the delivery of services being fragmented and inefficiently, sometimes duplicating services [6,7,8,9], from the viewpoint of the health system. Early in the 2000s, in response, decision-makers from all over the world made a commitment to “integrate” healthcare, initially concentrating on HIV and contraceptive services [10,11,12]. The focus of the conversation has moved over time to include both the integration of HIV and TB services and the integration of HIV and TB care into basic healthcare services [13,14,15,16]. Depending on the services and region, the meaning of integration may change globally. It could be used to describe services offered at the same facility by multiple providers or by the same provider within the same client–provider interaction [17,18,19,20,21]. Integration has long been a major area of policy concern in South Africa. Integration is viewed as a key strategy for providing effective and comprehensive care in the nation’s public health system and is featured as a concept in many national-level policy documents [22,23,24]. In order to improve health outcomes and the effectiveness of the healthcare system, the National Department of Health started “re-engineering” its public primary healthcare services in 2010 [25,26,27]. The country’s plan for integrated chronic disease management was then introduced shortly after, stressing the significance of “integration of care for patients with chronic communicable and non-communicable diseases such that patients are treated as individuals and not disease entities” [28,29,30,31]. The country’s services for sexual and reproductive health, TB management, and HIV care have benefited greatly from integration. The second-highest rate of TB incidence and the country with the highest proportion of HIV-positive people are both found in South Africa [18]. All active TB patients have HIV co-infection in about 57% of cases [30,31,32,33,34,35,36]. The President’s Emergency Plan for AIDS Relief (PEPFAR) of the United States provided funding to local NGOs to provide antiretroviral therapy (ART) in standalone facilities known as Comprehensive Care, Management and Treatment of HIV and AIDS sites, or CCMTs, when these services were first made available in the country along with widespread counseling and testing [37]. In contrast, the South African government soon assumed more accountability for health management, and during the years 2013–2014, stand-alone facilities were merged into public health facilities. The South African government supported 78% of HIV-related management initiatives in 2015 [38]. At public facilities today, nurse-initiated and controlled HIV care and treatment is the standard [39]. The goal in these situations is integrated management of HIV, TB, and other primary care services, including sexual and reproductive healthcare services. Yet, there is not enough scientific data to assess where and how integration is actually happening [40]. The patient’s perceptions both before and after the switch to integrated care have received very little attention. In order to understand why the integration of HIV and TB services into primary healthcare services occurred, what it looks like now, and potential implementation barriers, this study set out to investigate the opinions and experiences of patients.

## 2. Materials and Methods

### 2.1. Study Design and Period

This cross-sectional study was conducted applying a qualitative approach to explore perspectives on TB and HIV integration between May and June 2017. We reported our study findings using consolidated criteria for the Reporting Qualitative research (COREQ) checklist.

### 2.2. Study Setting

The study was conducted at Oliver Reginald (O.R) Tambo District Municipality in the Eastern Cape region of South Africa. The O.R. Tambo district has a total area of 12,141 km^2^ and an overall population density of 3800/km^2^. The racial makeup is as follows: Black African, 94.21%; Colored, 6.7%; Indian/Asian, 1.2%; and White, 2.69%, with isiXhosa the dominant language spoken by 85% of the people, followed by English, 8.6% [39]. It has a total population of 1,270,514 million, with Male 46.16% and Female 53.84%. The seat of OR Tambo is the city of Mthatha. The district is named after Oliver Reginald (OR) Tambo, an anti-apartheid activist and politician and a central figure in the African National Congress (ANC). The district code is DC 15 within the Wild Coast region. It is surrounded by the Alfred Nzo district (DC44) to the North, Sisonke district (DC43) to the North, Ugu district (DC21) to the northeast, Amatole district (DC12) to the southwest, Chris Hani district (DC13) to the west, and Joe Gqabi district (DC14) to the northwest. Local municipalities under O.R. Tambo district include Ngquza Hill, Port St. Johns, Nyandeni, Mhlontlo and King Sabata Dalindyebo [39]. The district is made up of four health sub-districts: King Sabata Dalindyebo (population 442,318), Mhlontlo (population 221,827), Nyandeni (population 436,813) and Qaukeni (population 659,431). O.R. Tambo district has been reported as having the following basic indicators: 64.6% of people live in poverty, the estimated unemployment rate is 65.5% and the literacy rate is 42.2% [39].

#### Study Sites

The study sites included the following primary healthcare clinics (PHC); Mbekweni [PHC number one], Gateway [PHC number two], Mhlakulo [PHC number three], Qumbu [PHC number four] and Libode [PHC number five]. Based on a review of their clinic records and reports from their catchment areas, the five health facilities mentioned above were purposefully selected across the O.R. Tambo district due to their high rates of TB and HIV patients compared to the other PHCs.

### 2.3. Study Population, Eligibility and Sampling

The study population consisted of these categories of patients; TB-infected, HIV infected and TB-HIV co-infected individuals. These patients were enrolled as study participants after informed consent was obtained. Eligibility criteria were such that those enrolled must be residents in the study area, aged 18 years and over, both male and female, and were willing to participate in the study. Furthermore, these participants must be those diagnosed with TB, HIV, TB-HIV co-infected and registered for care at any of the study sites. The TB and HIV patients were identified from the clinics register list provided by the nurses at the study sites. From these lists, eligible participants were purposively identified and enrolled in the study.

### 2.4. Data Collection Procedure

#### 2.4.1. Data Collection Tool

The data collection tool was an interview guide which comprised one central question, which guided the interviews, namely “what is your views about the TB and HIV integration in this facility?” Participants were probed to elicit more information from them, and the probing questions were dependent on participants’ responses to the central question. A pre-test discussion was carried out among five participants from the same facility to test the feasibility of the discussion topics or concepts if they were comprehensible to the study participants. Those five test participants were excluded from further participation in the main Focus Group Discussions (FDGs). Questions were modified as further insights emerged during data collection as studies by Addo et al. [3] indicated.

#### 2.4.2. Data Collection Process

The data were obtained through focus group discussions (FGDs) for consenting participants. We used focus groups rather than individual in-depth interviews because we thought they were the best way to exchange perspectives and have a more thorough exploration of any differences of opinion among participants, even though people discussed sensitive issues with caution due to stigma and discrimination. To ensure a conducive environment for privacy and prevention of plausible cross infection among study participants, the interviews were held in clinic consulting rooms with windows and ample space for all selected participants. These interviews were tape recorded and arranged so that FGDs were held separately for all TB-infected participants, HIV-infected participants, and co-infected (TB-HIV) participants. For each of the five participating PHCs, the community leaders mobilized and guided members of the community, facilitating problem solving and decision making. They also facilitated the FGDs but did not participate in the discussions so that participants who lived in the same community as the community leaders felt comfortable expressing their feelings about TB and HIV services. There were five (5) face-to-face focus group discussions (FGDs) with each comprising of at least six participants; two (2) TB-infected-only females, two (2) HIV-infected-only males, two (2) co-infected males and two (2) HIV-infected females. One participant refused to participate in the study due to stigmatization and discrimination. Each FGDs were fully participating because they were relaxed, free and express their views. On average, each FGD took about thirty to forty minutes. The FGDs were moderated by one of the authors who was also assisted by two study trained research assistants (male and female, with Master of Public Health degree) who had commendable experience in TB and HIV-related qualitative research. The interviews were mainly in the local language (IsiXhosa). During the interviews, notes were also taken to capture emotional atmosphere, gestures, and signs made by all participants for cross-referencing. In addition, a poster of TB and HIV was introduced in the middle of the FGDs to stimulate participants for reactions and responses. This stimulation made participants expand on points made by others in the group. These interactions with different people in the discussion specifically brought out various opinions and great experiences that might not emerge during semi-structured interviews. As a result, information was forthcoming during the discussion as we continued to ask probing questions.

### 2.5. Data Management and Analysis

The audio data were recorded, translated from the native tongue to English, and then literally transcribed. Qualitative content analysis was used to examine the transcribed material (QCA). Prior to combining some of the related components, the authors carefully read and evaluated the focus group transcripts to determine meaning. Throughout the data collection procedure, we conducted ongoing analysis. With the same findings that ceased to offer new information after the completion of five FDGs, we had a strong and valid grasp of the research phenomena. In our opinion, five FGDs were enough to reach the saturation point and accomplish the study’s goals. The analysis employed no theoretical frameworks or models. For our data analysis, we employed Nvivo, version 9 of qualitative software.

### 2.6. Trustworthiness

According to Uebel et al. [21], “trustworthiness” or “rigor” refers to the study’s credibility, transferability, dependability, and confirmability. Credibility, reliability, conformability, and transferability were all taken into consideration to make sure that trustworthiness was upheld. Prolonged participation and member vetting ensured credibility. An independent coder was brought in to assure consistency, further boosting dependability, after experts were utilized to confirm the process. Transferability was made possible by gathering comprehensive data and writing concise descriptions of the facts. Notes were kept in a secure area to guarantee conformability, enabling the construction of an acceptable trail and the choice of conclusions and interpretations.

### 2.7. Human Subject Protection

Prior to the start of the study, research analysts who were directly involved in the recruitment of study participants underwent the National Institutes of Health (NIH) course on protecting human research participants, which is a requirement for everyone involved in studies involving human subjects.

### 2.8. Definitions of the Operational Concepts

Stigma is the negative stereotype and discrimination is the behavior that results from this negative stereotype [21].

Transferability refers to the degree to which the results of qualitative research can be generalized or transferred to other contexts or settings [21].

Conformity is defined as a type of social influence that elicits a change in belief or behavior to fit in [21].

Credibility is a measure of the truth-value of qualitative research or whether the study’s findings are correct and accurate [21].

Dependability refers to the consistency and reliability of the research findings and the degree to which research procedures are documented, allowing someone outside the research to follow, audit, and critique the research process [21].

Rigor is a way to establish trust or confidence in the findings of a research study. It allows the researcher to establish consistency and accurate representation of the population studied [21].

Qualitative content analysis involves a process designed to condense raw data into categories or themes based on valid inference and interpretation [21].

Pneumocystis pneumonia is a type of infection of the lungs (pneumonia) in people with a weak immune system [1].

## 3. Results

### 3.1. Description of the Study Participants

Of the 29 participants of the study, 10 were males (Table 1). The highest number of the participants were married (9, 31.3%) and widowed (9, 31.3%). The majority of participants had a tertiary level of education (55%), which is representative of the purposely selected study group, followed by secondary levels of education (45%). In addition, the study population predominantly were black and all were unemployed (Table 1).

### 3.2. Themes

Out of the 29 participants, 24 gave information that is related to theme one (1), two (2), three (3) and four (4). Four (4) participants were added after data saturation to confirm that there was truly nothing new. In seeking to identify knowledge gaps, conceivable barriers, and facilitators of TB-HIV integration services at the facilities from the patients’ perspectives, the researchers uncovered three themes, each with one to four subthemes. Table 2 presents a summary of themes and sub-themes that emerged from data analysis. These themes revealed the extent of knowledge on TB, HIV, TB-HIV co-infection, and the management thereof. A narrative interpretation of each of these themes and subthemes are discussed in the subsequent subsections.

#### 3.2.1. Theme 1: Knowledge of Risk Factor for TB, HIV and TB-HIV Coinfection Etiology of Tuberculosis (TB)

The participants’ perception indicated that there is gap on understanding the risk factors for TB and/or HIV. In addition, they did not understood a causal relationship between TB and HIV, as this shown by participants who indicated that people with HIV should not be concerned with acquiring TB. This perception by participants has suggested that healthcare workers have to consistently promote health education to enhance understanding among TB, HIV and co-infected patients including causal link. The lack of knowledge about the TB and its relationship to HIV disease cause an underutilization of the health services and a delay in testing TB in HIV-positive patients who are at risk of acquiring TB and then need to be put on cotrimoxazole for TB-preventive therapy. Cotrimoxazole preventive therapy (CPT) is recommended for the prevention of morbidity and mortality due to pneumocystis pneumonia and other infections in HIV-positive patients with low immunity. Improving the roll out of TB and HIV public awareness and health promotion is important in increasing knowledge about TB and HIV. This was the narrative from participants (coded Clinic Two on Male Patient on HIV medication):

*“I do not think you should be concerned about TB when you are HIV positive because you have medication.”* #1

This was emphasized by another participant (Female Patient on HIV medication) who said:

*“I am on my ARVs myself and I am also not concerned or worried about TB as I am compliant patient to my medication and I do not understand why would you worried about TB cause for me TB affects smoking people not people who are taking ARVs.”* #2

#### 3.2.2. Theme 2: TB-HIV Integration Management Services at the Healthcare Facility

With regard to how well TB-HIV services were integrated at the healthcare facility, the study participants were unaware of the existence of integrated TB-HIV management services at the facility. The study participants did not understand the integration and how it should be defined and implemented in public health facilities. The researcher assistants outlined that when services are fully integrated, patients need not to return to the clinic on different days for different services, stand in more than one queue when they visit, or see more than one healthcare provider during a visit. After the explanation, participants believed that some services of TB and HIV are available but at times, some patients have to be referred to other PHCs for other TB services. The study showed that TB-HIV integration management is still inadequate, and there is a disconnection between the programs (partial integration), which meant an increase in the cost of care for patients, as well as other added inconveniences, as numerous visits were required to access the required care. Direct quotes from the participants that conform to the narrative for this theme are presented below.

*“The way I see it, both TB and HIV are available because I have tested for TB before here, however, at times I will be referred to another clinic because of some of medication is not available or I go to the nearest TB hospital for my TB medication.”* Clinic One, Male Patient on HIV-TB medication #2

This was supported by another participant (Female Patient on TB-HIV medication) who said:

*“I have quite similar problem where I have to go to the hospital for bloods of viral suppression of which it cost me to go to hospital and make those long queue, the clinic is not far from my home, but unfortunately it is not providing the service and they only do HIV testing in this clinic.”* #3

##### Diagnostic Process

According to our study findings, nearly half of those who had a confirmed TB diagnosis did not receive HIV testing or counselling as can be seen in the participant quotes below. This might be inconsistency in testing and counselling by healthcare workers. It is highly recommendable for TB patients to consistently test for HIV, as HIV weakens the immune system for opportunistic infections such as TB.

*“I coughed for sputum, but I never took bloods for Voluntary Counselling and Testing (VCT), maybe I am still going to take bloods nothing has been said about voluntary testing and counselling so far.”* Clinic Two, Male Patient on TB medication #4

This was emphasized by another participant (Male Patient on TB medication) who said:

*“For me I coughed sputum, but I was told to comeback for VCT and I am asking myself why the nurses should not do that VCT when I am here for the medication at least.”* #5

##### Dissemination of Information/Counseling

As an indicator of TB-HIV integration, the study observed that majority of the participants did not receive information or counseling about antiretroviral drugs (ARVs), HIV, nutrition, tuberculosis or other preventive measures. There might be a lack of consistency in health education programs on TB-HIV by the healthcare workers. Programs of TB-HIV control management place emphasis on treatment adherence especially awareness of side effects, a well-balanced, behavior alteration in sex life (condom use, abstinence, be faithful, reduction in multiple partners by patient), lifestyle changes (such as reduction smoking and alcohol intake) and adherence support strategies services may include directly observed treatment.

*“I have never received a good health education on ARVs, HIV and TB, nutrition and prevention strategies, however I have been told about vegetables and condom use but I think there is a gap as I struggle with my medication side effects sometimes since I just started in a month ago especially TB medication with nausea and vomiting after taking the pills.”* Clinic Three, Female Patient on TB-HIV medication #5

##### Infection Control Practices against TB and HIV

Based on the TB-HIV integration guidelines, we explored whether or not the facility employed an infection prevention and control (IPC) nurse for implementation of policies, knowledge and education for the facility. The majority of respondents were not aware about the existence of infection control nurse in their facility, which is an indication that facility have sub-optimal infection control measures in place, and it is also an indication that the facility has challenges regarding the implementation of effective IPC programs. This was also a narrative from the patient (Clinic Four, Female Patient on TB medication)

*“I have not seen an infection control nurse since I started taking my treatment at this clinic, this is my fourth month now, however I usually see different people who assist with triaging system and usually the admin staff help or security.”* #6

This was emphasized by another participant (Male Patient on TB medication) who said:

*“I can agree with him also, I have not seen an infection control nurse other admin people who are always triaging patients, I am on my sixth month taking my medication, however, the other day I came we had health education on how to wash hands and putting hands on the mouth when coughing.”* #5

#### 3.2.3. Theme 3: Factors Affecting Compliance

We explored the factors that could cause non-compliance with treatment protocols. One of the factors that emerged was the long distances between respondents’ homes and the healthcare facilities, which prevented respondents from seeking care as planned. The study observed that the majority of respondents were concerned about long distance to clinics, poor quality roads and transportation costs, which negatively affected their accessibility for TB and HIV services. Infrastructural challenges and distances are barriers to the facility for accessibility TB and HIV services especially in disadvantaged communities. This was a narrative from the patient (Clinic Four, Male Patient on HIV medication)

*“Distance for me to this facility is still a problem, at times it is gets dark while I am still travelling home, since the clinic is far from where I stay. But I try to come, since my health is important even roads are very bad especially when it is raining.”* #2

This was supported by another participant (Female Patient on HIV medication) who said:

*“I am also experiencing the same problem whereby I arrive late and by the time I am at home its dark, at home they always ask me why I always come late when visit the clinic so I tell them that distance to the clinic is the problem.”* #3

#### 3.2.4. Patients’ Recommendations for Improvement of TB-HIV Integrated Healthcare Services

##### Perception on Quality of Service

When asked whether the clinic’s quality of TB and HIV care was good, the majority were not happy with the services rendered by the facility. Most respondents thought that their facility’s TB and HIV services were of very poor quality. This could be a result of inconsistent management of the integration of TB and HIV. This was a narrative from the patient (Clinic Four, Male Patient on TB-HIV medication)

*“Quality is still poor. We have been told about vegetables like spinach and cabbage and condom use, but we need a good health education on TB and HIV, nutrition and other prevention strategies of TB and HIV.”* #3

This was emphasized by another participant (Female Patient on TB-HIV medication) who said:

*“For me, quality means that TB and HIV are both available, I can access them here in this clinic without going to other facilities, because it has cost me to be diagnosed in this facility, then been told that some of my medication is not available and be referred to the hospital.”* #4

##### Patients’ Recommendations for Improvement

When asked if they believed the quality of care provided by their facility could or should be enhanced, the majority of respondents agreed that service could be improved. It therefore appears that TB and HIV services are negatively perceived by most patients and could be improved. It is possible that those who found the services adequate were not frequent clinic attenders, being affected by the distance between the facility and their homes. Their responses may also have been influenced by their inability to compare the quality of services available, being unable to afford to travel to alternative facilities. This was a narrative from the patient (Clinic Four, Female Patient on TB-HIV medication)

*“I recommend that services of TB and HIV in our facilities should be improved, with full integration (one stop shop) model of TB and HIV services, so that we can access both services where I am are staying, without travelling distances, because I am are currently not employed but us and cover our cost to these other facilities.”* #5

This was supported by the whole FDG (clinic four) who said:

*“We indeed not happy with the current model of TB and HIV services in our facility, we are appealing to district and department of health that should improve the services so that we are all have access to quality TB-HIV integrated services travelling from where we are staying to other facilities is not ideal.”* #4

## 4. Discussion

The exploration of patients’ perceptions of the integration of tuberculosis and HIV/AIDS services in primary healthcare facilities in the O.R. Tambo District of the Eastern Cape province of South Africa indicated that patients had sub-optimal knowledge of the risk factors for TB and/or HIV. These findings are similar to those of a study conducted in South Africa [5], where about 60.2% patients knew that TB is spread by bacteria but very few had an adequate knowledge of TB. Furthermore, our study found poor compliance with the guidelines in that most HIV patients reported not being screened or tested for TB, and vice versa for TB patients, who reported not being counselled or tested for HIV. This is indicative of no integration or partial integration, in which some HIV services are provided in general clinics and some TB services are provided in HIV clinics, but co-infected patients still need to visit two separate clinics served by different staff to access the full range of HIV and TB services. These findings are similar to those of other studies conducted in South Africa and other African countries [5,7,8,9,10,11,12,13]. According to the guidelines for the integration of TB and HIV services, HIV testing and counselling must be performed for patients with presumptive and diagnosed TB. Similarly, HIV patients should be screened to rule out tuberculosis and begin tuberculosis prevention with isoniazid preventive therapy and early antiretroviral therapy [6]. In addition, our study found that most respondents did not receive adequate and comprehensive education on ARVs, HIV, diet and TB, a knowledge of which is a critical facilitator of good clinical outcomes among co-infected patients. It is possible that this inadequate provision of services is a consequence of program inconsistency, in which some people receive the full range of services and others do not. Health education is crucial to enabling patients to comply with their treatment, and it is typically integrated into services provided at the primary healthcare (PHC) level [2]. Health education is also stipulated as a guideline for the integration of TB and HIV services [6,10] (weblink above). There have been concerns about the patient–doctor relationship in public healthcare, particularly as it relates to health literacy. The education of patients about their health continues to play an important role in fostering a positive relationship between patients and healthcare providers, which in turn facilitates adherence to treatment protocols and increases follow-up among TB-HIV patients [15,16].

Another disturbing finding from our study was the lack of infection control enforcement staff in the facilities. This is concerning because infection is known to complicate clinical care, increase length of stay in hospitals, and have a disruptive effect on patients’ recovery and a devastating effect on healthcare facilities, especially in low resource settings [17,18]. A paucity of infection prevention and control experts, trained in the field, also confirms the inadequacy of the management and enforcement of infection prevention control guidelines, which puts patients at risk [19]. The presence of positive proactive leadership, sufficient and competent support staff, and infection control coordinators in every healthcare facility is essential for the effective control of infections in healthcare institutions [20,21,22,23]. In ideal conditions, all of the above would form part of a committed team, with each member having clear responsibilities and boundaries. Our study noted that the majority of respondents believed that the quality of TB and HIV services was still inadequate in their facilities. These findings reflect poor TB-HIV service integration. It is unsurprising that many respondents mentioned poor satisfaction with the quality of services they received. This is supported by evidence on the TB care cascade, which shows that over 25% of TB cases do not access government TB facilities or have never sought TB care [11]. According to UNAIDS (22), quality TB-HIV services include: (i) screening and evaluation for TB and HIV with appropriate tests, access to prevention education for TB and HIV among those who screen negative, and linkage to appropriate treatment for TB and HIV; and (ii) timely identification of both HIV and TB, and linkages to appropriate treatment (full integration). The literature has demonstrated that nearly 12 million TB-related HIV deaths were averted in the period 2000–2020 because of TB/HIV-related interventions (i.e., full integration of services) [24,25,26,27]. Hence, the integration of TB-HIV care is a predictor of good TB and HIV treatment outcomes, as is adequate, strong management and leadership at the facility level, as other studies have shown [28,29,30,31]. Findings by Kanyerere et al. [24] in Kenyanian clinics show that patients observed a lack of quality in TB-HIV services at their facilities, while in Durban, a South Africa Health Systems Trust study highlighted similar findings [25]. Studies in other countries have made similar observations: Fernandez-Lazaro et al. [31] in Brazil, Owiti et al. [32] in Kenya, and Obaromi et al. [33], Hassan et al. [34] and Kigozi et al. [5] in South Africa. The current study explored whether long distances to healthcare facilities prevented respondents from seeking care as planned. This was the case for 75.9% of respondents. Thus, one may conclude that for the majority of patients, the challenge of distance is a preventative to adequate access to TB and HIV services. The World Health Organization (WHO) [1] has emphasized that in order to maintain accessibility, primary healthcare facilities must be situated within five kilometers of the home of any member of the population. Studies conducted by Paramasivan et al. [36] in Kerala, Sinshaw et al. [37] in Ethiopia and SANAC [38] in South Africa have shared similar findings. Statistics South Africa characterizes the O.R. Tambo district as poor, with many challenges in terms of socio-economic status and unemployment, both of which affect access to TB-HIV services that would promote adherence and control of TB and HIV [39]. Addo et al. [40] and Sathar et al. [41] made similar findings with regard to patients in low-income countries, showing that poor patients tend to miss appointments because of distances and lack of money to honor their clinic visits. Studies in Malawi, Botswana, Democratic Republic of Congo, and Kenya have demonstrated similar findings [24, 26]. Our study confirms these studies, showing that the majority of respondents are unhappy with the current level of HIV-TB healthcare integration in O.R. Tambo district clinics. The strong recommendation from study participants was that the quality of services offered be improved. Participants’ suggestions for improvements are in line with the stipulations of UNAIDS [27,28] and WHO [42,43,44,45]. These organizations urge sub-Saharan countries to make the best use of their limited resources rather than use lack of resources as a justification for poorly integrated TB and HIV control programs. The differences in the perception of health services detected among TB, HIV and TB-HIV were almost the same to all the participants and perceived service delivery of TB and HIV inadequately; maybe it could be because of the current service delivery model of TB and HIV management in the district, which the majority of the study participants perceived as lacking quality. The World Health Organization (WHO) has recommended the package of interventions collectively called “collaborative TB/HIV activities” as a key strategy in reducing TB-related deaths among people living with HIV, and these recommendation were made to address and improve quality of care for both TB and HIV services [45]. HIV-TB services refer to screening, diagnosis, and treatment services provided for both diseases at the same clinic, by the same clinic team, on the same visit day, according to the World Health Organization. Similar studies elsewhere have reported that an integrated therapy of both HIV and TB based on the current evidence of studies from both diseases has been shown to be feasible and efficient in controlling the diseases and yields better survival in various clinical settings [2,19,32,43,45]. This study was conducted in a district that is mainly influenced by the high rate of poverty and overburdened by co-infection [33], owing to the size of the study; our results can only be representative of the O.R. Tambo District Municipality.

### Study Limitations

In our study, we employed a qualitative approach to explore the participants’ perceptions on the integration of HIV, tuberculosis and primary healthcare services in O.R. Tambo District. Study participants were included from only one municipality; participants from other areas might have different perceptions about TB-HIV integration that might influence the study findings. We also used only the FGDs method to find out an in-depth understanding of social issues; therefore, there was no methodological triangulation, and we could not overcome the intrinsic bias. Even though most participants were females and had post-secondary education, these numbers only represent the sample population that was intentionally chosen for the study. Moreso, the study’s participant gender and educational levels would not have any difference or impact because according to our study findings, the healthcare system is failing the patients. For strong empirical evidence, we suggest that future researchers repeat this study using a mixed-methods approach.

## 5. Conclusions

Overall, most of the participants, who were all patients from the clinical sites in our study, had limited knowledge of TB; most, for example, did not know that tuberculosis (TB) spreads through the air or that it is linked to the human immunodeficiency virus (HIV). Their lack of knowledge may well have been influenced by infrequent visits to the clinics, since most reported being affected by long distances and lack of transportation. From the health systems, inadequate information sessions to enlighten patients about TB and HIV disease and management could also contribute to the lack of knowledge identified. In addition, studies elsewhere have observed that the healthcare system can be a barrier to the integration of TB-HIV due to the factors such as staff shortages in the clinics, insufficient training and experience, outdated clinic infrastructure, and policies not being implemented [19,29,44,45]. Some studies reported concerns about the impact of integration on staff workloads and waiting times. Finally, there were concerns about TB integration due to infection control issues [2,3,4,45,46,47]. Furthermore, this study found that the district healthcare facilities lacked infection control coordinators and had not taken appropriate measures to address the problem. There was also a concerning lack of HIV testing for TB patients and vice versa for HIV patients. Without further ado, the findings highlight areas that require additional research to resolve the problems plaguing the TB and HIV control program so that relevant stakeholders in the study setting, the country, and other TB-HIV-endemic settings can address these issues effectively.

## Figures and Tables

**Table 1:** Characteristics of the study participants in O.R. Tambo District Municipality.

	Variable	Frequency (*n* = 29)	Percentage (%)
	18–29	7	24.13
	30–39	7	24.13
Age	40–49	4	13.79
	50–59	7	24.13
	Over 60	4	13.79
Population group	White	2	6.89
	Black	27	93.10
Gender	Female	19	65.52
	Male	10	34.48
	Married	9	31.03
Marital status	Single	7	24.14
	Living with a supporter/partner	00	00
	Widowed	9	31.03
	Divorced	4	13.79
	No schooling	00	00
Education level	Primary	00	00
	Secondary	13	44.83
	Tertiary	16	55.17
Religion	Christianity	29	100
	Employed	00	00
Occupation	Self-Employed	00	00
	Unemployed	29	100

**Table 2:** Patients’ perceptions about the quality of integration of HIV, tuberculosis and primary healthcare services in OR Tambo District Municipality.

Themes	Subthemes
Knowledge of risk factors for TB, HIV and TB-HIV coinfection	Sub-optimal TB knowledge
TB-HIV integration management services at the healthcare facility	Observed TB-HIV service integration practices at the facility
-	The diagnostic process
TB-HIV integration management services at the healthcare facility	Dissemination of information/counselling
Infection control practices against TB and HIV	Observed lack of infection control coordinator at the facilities.
Factors affecting compliance with treatment protocols	Respondents’ perceptions of quality of service
Patients’ recommendations for improvement of TB-HIV integrated healthcare services	Proposed improvement strategies

## Data Availability

The data presented in this study are available on request from the corresponding author. Full data are not publicly available due to privacy restrictions.
